# Bioinformatic identification of candidate biomarkers and related transcription factors in nasopharyngeal carcinoma

**DOI:** 10.1186/s12957-019-1605-9

**Published:** 2019-04-01

**Authors:** Zhimin Ye, Fangzheng Wang, Fengqin Yan, Lei Wang, Bin Li, Tongxin Liu, Fujun Hu, Mingxiang Jiang, Weiyang Li, Zhenfu Fu

**Affiliations:** 1 Department of Radiation Oncology, Zhejiang Cancer Hospital, Hangzhou, Zhejiang China; 2 Key Laboratory of Head & Neck Cancer Translational Research of Zhejiang Province, Hangzhou, Zhejiang China; 3 Department of Radiology, Zhejiang Cancer Hospital, Hangzhou, Zhejiang China

**Keywords:** Nasopharyngeal carcinoma, Differentially expressed genes, Bioinformatic analysis, Transcriptional factors

## Abstract

**Background:**

The incidence of nasopharyngeal carcinoma (NPC) is rare, but a certain amount of mortality remains in NPC patients. Our study aimed to identify candidate genes as biomarkers for NPC screening, diagnosis, and therapy.

**Methods:**

We investigated two microarray profile datasets GSE64634 and GSE12452 to screen the potential differentially expressed genes (DEGs) in NPC. Gene ontology and Kyoto Encyclopedia of Genes and Genomes (KEGG) pathway enrichment analysis of the DEGs were also performed. A protein-protein interaction (PPI) network of DEGs was constructed by STRING and visualized by Cytoscape software. The associated transcriptional factor regulatory network of the DEGs was also constructed.

**Results:**

A total of 152 DEGs were identified from the GSE64634 and GSE12452 datasets, including 10 upregulated and 142 downregulated genes. Gene functional enrichment analysis indicated that these DEGs were enriched in the cilium movement, antimicrobial humoral response, *O*-glycan processing, mucosal immune response, carbohydrate transmembrane transporter activity, hormone biosynthetic process, neurotransmitter biosynthetic process, and drug metabolism-cytochrome P450 pathway. Five hub genes (DNALI1, RSPH4A, RSPH9, DNAI2, and ALDH3A1) and one significant module (score = 5.6) were obtained from the PPI network. Key transcriptional factors, such as SPI1, SIN3B, and GATA2, were identified with close interactions with these five hub DEGs from the gene-transcriptional factor network.

**Conclusions:**

With the integrated bioinformatic analysis, numerous DEGs related to NPC were screened, and the hub DEGs we identified may be potential biomarkers for NPC.

## Background

Nasopharyngeal carcinoma (NPC) is the most metastatic cancer of the head and neck that possesses a unique geographical distribution and ethnic populations. There were an estimated 129,000 new cases and 73,000 deaths in 2018, with approximately 92% of new cases occurring in less economically developed countries [1]. Compared with other cancer types, NPC is characterized with several well-defined populations, with the highest incidence taking place in Southeast Asia, Micronesia/Polynesia, Eastern Asia, and Northern Africa, but rarely been reported in Americas and Europe [2]. Except for the implication of environmental factors in the etiology of NPC, Epstein-Barr virus (EBV) has been proved to be a convincing risk factor for the pathogenesis of NPC [3, 4]. Intensity-modulated radiation therapy (IMRT) is the primary treatment for NPC and has favorable outcomes with nearly 80–90% curative rate in the early stage, and the overall 5-year survival rate for all NPC stages ranges from 50 to 70% [5]. Nevertheless, more than 70% of patients with NPC are diagnosed with advanced stage disease, and almost half of these patients experience poor prognosis [6]. Moreover, distant metastasis is the typical characteristic for NPC and the tumor is often found to metastasize to lymph nodes as well as to distant organs such as the brain and lung by the time of diagnosis, which is still the main cause of treatment failure [7]. Furthermore, therapeutic resistance and toxic effects also pose serious threats to the prognosis of NPC.

With the rapid development of gene chip and RNA sequencing technologies, bioinformatic analysis plays a significant role in screening candidate biomarkers for various diseases especially cancers [8]. Bioinformatics provides novel clues and core data for identifying reliable and functional differentially expressed genes (DEGs), microRNAs (miRNAs), circular RNAs (circRNAs), and long non-coding RNAs (lncRNAs). Emerging evidence also demonstrates the critical role of bioinformatic analysis for precise screening, prompt diagnosis, and molecular-targeted treatment in various types of cancers [9–11]. Great effort also has been put into the screening biomarkers for the diagnosis of NPC, such as EBV DNA and lactate dehydrogenase (LDH), but other candidate biomarkers with the ability to predict tumor progressions still need to be identified to guide clinical treatment for NPC patients.

In the present study, the DEGs between normal nasopharyngeal tissues and nasopharyngeal carcinoma tissues were screened from the microarray expression profiles based on the Gene Expression Omnibus (GEO) database. Gene ontology (GO) and Kyoto Encyclopedia of Genes and Genomes (KEGG) pathway enrichment analysis were also performed for the functional analysis of DEGs. A protein-protein interaction (PPI) network of DEGs was constructed by STRING and visualized by Cytoscape software. Furthermore, a gene-transcriptional factor (TFs) regulation network of hub DEGs was constructed to assess the interactions between the TFs and hub DEGs. Based on the bioinformatic analyses, our study is expected to provide novel insights into the NPC and to identify NPC-associated DEGs as potential biomarkers for disease prognosis.

## Materials and methods

### Microarray data information

The GEO database, which is a public functional genomics data repository including array- and sequence-based data of gene profiles and next-generation sequencing, was applied for the NPC mRNA expression profiling studies. The keyword “nasopharyngeal carcinoma” was inputted to search for the suitable dataset, and we only selected the original microarray studies that analyzed mRNA expression profiling between normal nasopharyngeal tissues and NPC tissues in *Homo sapiens*. Two mRNA microarray datasets GSE64634 and GSE12452 were selected from the GEO database. The GSE64634 dataset was based on the GPL570 platforms ([HG-U133_Plus_2] Affymetrix Human Genome U133 Plus 2.0 Array) and included 4 normal nasopharyngeal tissues and 12 nasopharyngeal carcinoma tissues. The GSE12452 dataset was based on the GPL570 platforms and included 10 normal nasopharyngeal tissues and 31 nasopharyngeal carcinoma tissues.

### Data processing and DEG identification

The raw microarray data of the two datasets downloaded from the GEO database were processed by the online tool GEO2R (http://www.ncbi.nlm.nih.gov/geo/geo2r) to identify genes that are differentially expressed between normal nasopharyngeal tissues and nasopharyngeal carcinoma tissues. The results are presented as a table of genes ordered by significance (Table 1), and a |log fold change (FC)| > 2.0 and *P* value < 0.05 were further conducted as the cutoff criteria for the DEG screening. Subsequently, the overlapping DEGs between GSE64634 and GSE12452 were identified based on the online tool Venny 2.1.0 (http://bioinfogp.cnb.csic.es/tools/venny/index.html). The heatmap of DEGs was drawn by the web-based Morpheus software (https://software.broadinstitute.org/morpheus/).

**Table 1:** One hundred fifty-two differentially expressed genes (DEGs) were screened from GSE64634 and GSE12452 microarrays for nasopharyngeal carcinoma

DEGs	Genes symbol
Upregulated (10)	FN1, CXCL10, LAMB1, LHX2, CHI3L1, ZIC2, TNFAIP6, GAD1, NUF2, PARPBP
Downregulated (142)	SCNN1A, CCDC173, AGR2, GSTA3, FAM3D, PRSS23, UBXN10, SLC2A10, LRRC10B NME5, LOC100653057, CFAP53, C9orf116, TPPP, LOC101930373, DNAH9, ADGB, CCDC181, ZMYND10, OSCP1, SPATA17, KCNRG, CDHR3, CCDC96, CFAP70, ABCA13, MFSD4A, TMEM45B, IQCD, TMC5, NQO1, LRRC34, DUOX1, VWA3B, NEK5, CXCL17, CAPS2, IQCA1, RSPH9, CAPS, TTC9, SLC44A4, MS4A8, AKAP14, ADH1C, RBM24, ARMC4, ADGRF1, AK7, MDH1B, TMEM231, C11orf97, ARMC3, CFAP45, CATSPERD, C2orf40, SORBS2, PACRG, DNAI2, CHL1, WFDC2, SPAG6, MORN5, CCDC190, PIFO, CLU, TEKT1, MUC5AC, DNAJA4, DHRS9, CLDN23, CDH26, C4orf22, CFAP126, TTC29, ST6GALNAC1, CLDN10, MUC1, FAM216B, TMEM190, LRRC46, MIR675, RSPH4A, LOC101928817, AKR1C3, LOC101927416, F3, SERPINB3, ZBBX, SLC6A14, TTC25, ROPN1L, DNALI1, BPIFB1, TMEM232, CASC1, RRAD, SCIN, SAXO2, CFAP43, TSPAN1, SLPI, ALDH3A1, C11orf70, CAPSL, DNAH12, CHST9, CLIC6, TPPP3, KCNE1, CCDC113, CYP4B1, MUC16, CEACAM5, MSMB, SCGB1A1, DYNLRB2, ERICH3, UPK1B, MUC4, LCN2, C9orf135, CFAP52, C7orf57, CEACAM6, S100P, LOC101927057, EFHB, OMG, RSPH1, FAM81B, AGR3, PIGR, C9orf24, LTF, C19orf33, GSTA1, AQP3, CDC20B, C20orf85, SNTN, CES1

### Gene functional and pathway enrichment analysis of DEGs

The Metascape (http://metascape.org/) is an online analytical tool used to extract comprehensive biological information associated with large candidate gene lists. It can not only provide the typical gene-term enrichment analysis, but also visualize genes-term relationships, search for interesting and related genes or terms, and dynamically view genes from their lists on biological functions, pathways, and more. GO analysis and KEGG pathway analysis of the screened DEGs were carried out based on the Metascape tool. *P* value < 0.01 was set as the cutoff criterion, and significance was ranked by enrichment score (− log 10 (*P* value)).

### PPI network construction and identification of hub genes

The search tool for the retrieval of interacting genes (STRING, http://string-db.org) is a web-based resource for evaluation of the comprehensive information of the proteins and prediction of PPI for the input gene list. In this study, STRING was employed to gain information for the PPI of the DEGs, and each PPI pair with a reliability threshold of a combined score of > 0.4 was selected as significant interaction pair. Then, according to the interaction pair information, the PPI network was constructed and visualized by Cytoscape software (version 3.4.0, http://www.cytoscape.org/). The Cytoscape plug-in Network Analyzer was used for further analysis, and the topological properties of the PPI network, node degree, were calculated to search for hub genes from the PPI network. Subsequently, Molecular Complex Detection (MCODE) analysis in Cytoscape was performed to screen the significant modules of PPI network with node score cutoff = 0.2, K-Core = 2, and degree cutoff = 2 set as the cutoff parameters.

### Transcriptional factor regulatory network of hub genes

NetworkAnalyst (http://www.networkanalyst.ca/faces/home.xhtml) is a comprehensive web-based tool for network-based visual analytics of gene expression profiling, meta-analysis, and interpretation. It can support the integrative analysis of TF-gene interactions for the input genes and assess the effect of the TF on the expression and functional pathways of the hub gene. In this study, the TFs of the hub genes were predicted from this database and a gene-TF regulatory network was constructed and visualized by the Cytoscape software.

## Results

### Identification of the DEGs of NPC

**Fig. 1:**
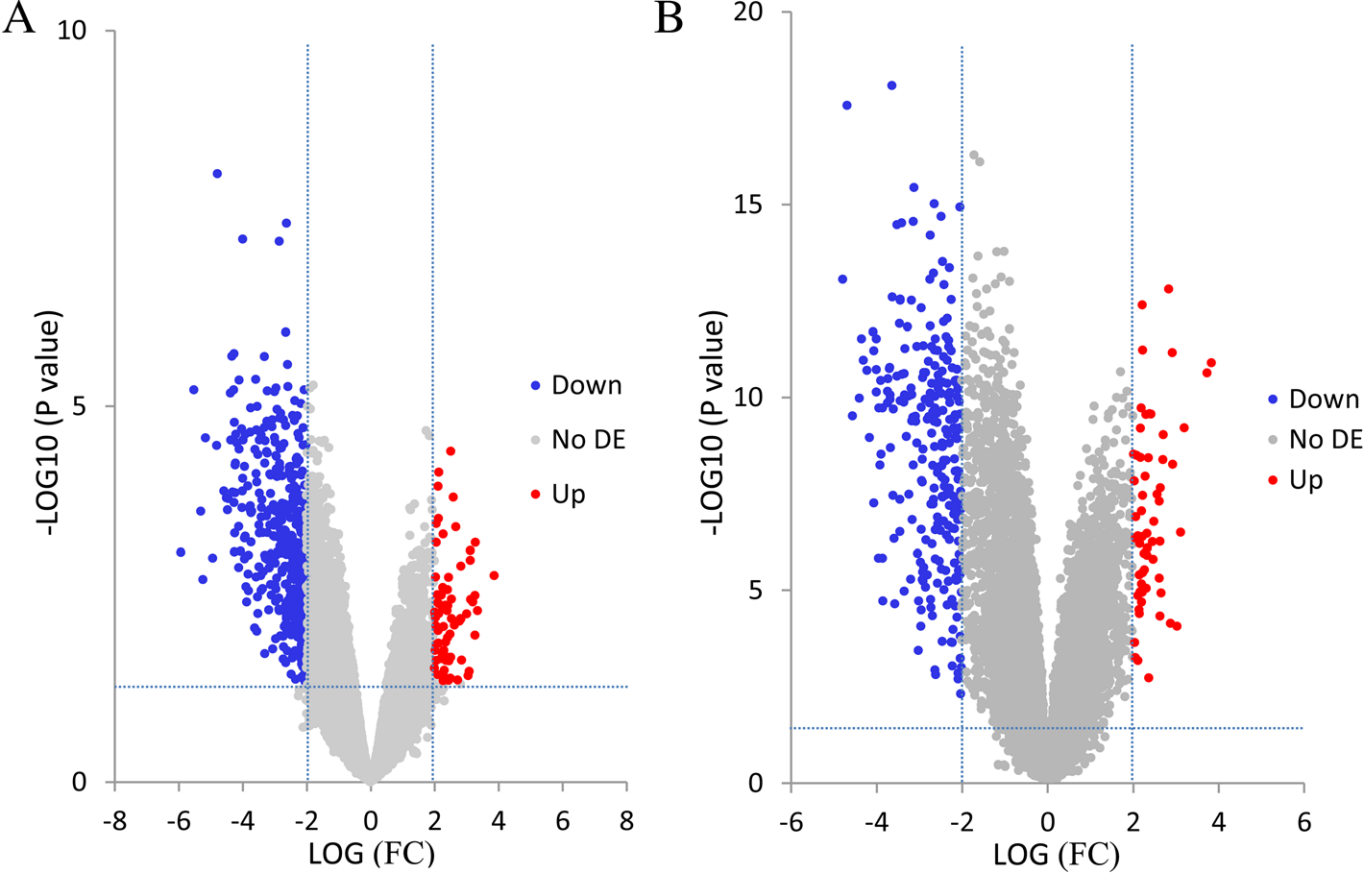
The volcano plots of DEGs in GSE64634 (**a**) and GSE12452 (**b**) microarrays. Red indicates genes with high levels of expression, blue indicates genes with low levels of expression, and gray indicates genes with no differential expression based on the criteria of *P* value < 0.05 and |log FC| > 2.0, respectively

**Fig. 2:**
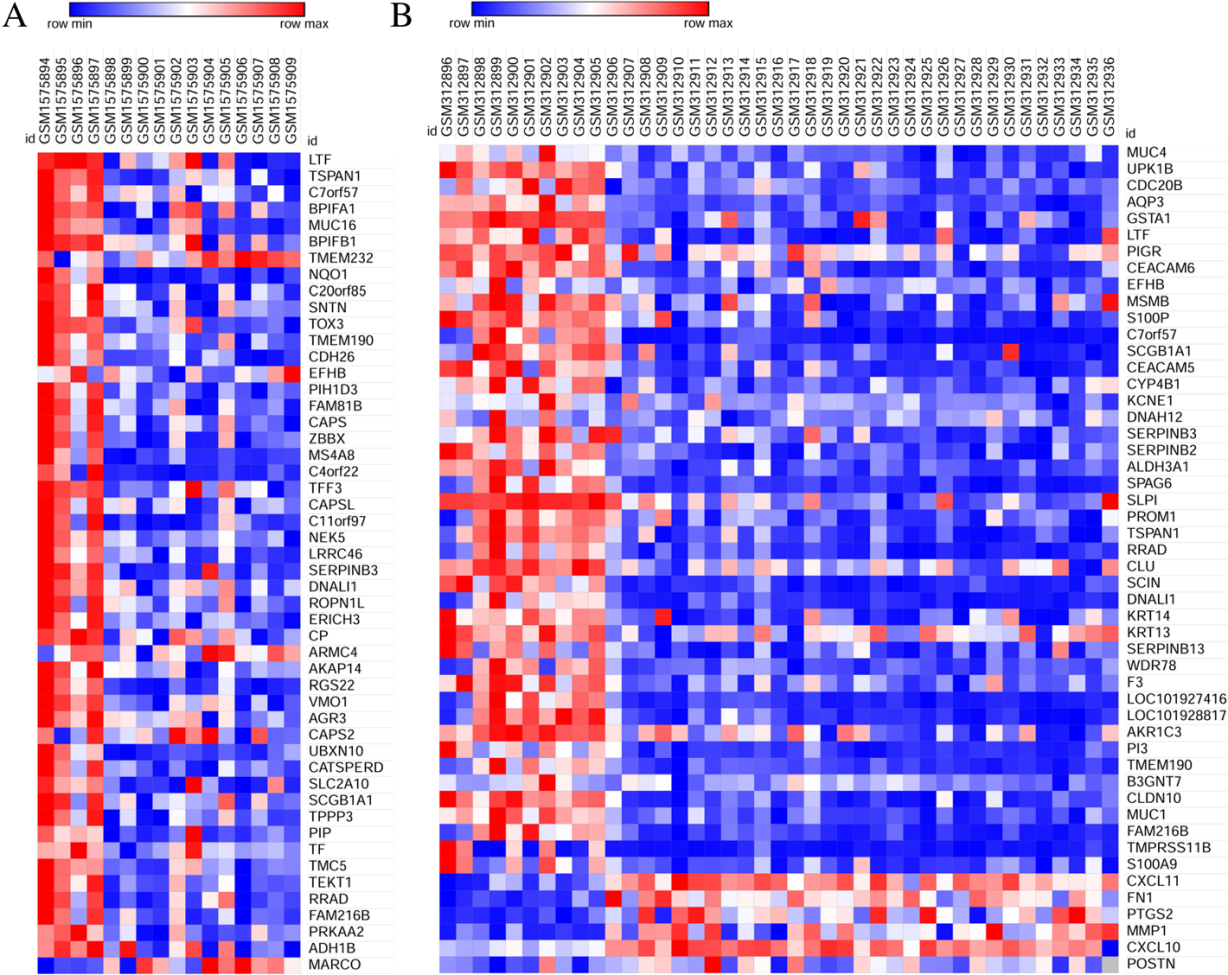
Heatmap of top 50 differentially expressed DE mRNAs in GSE64634 (**a**) and GSE12452 (**b**) microarrays. Gene expression levels were indicated by colors as shown by the row, red represents high expression level and blue represents low expression level

**Fig. 3:**
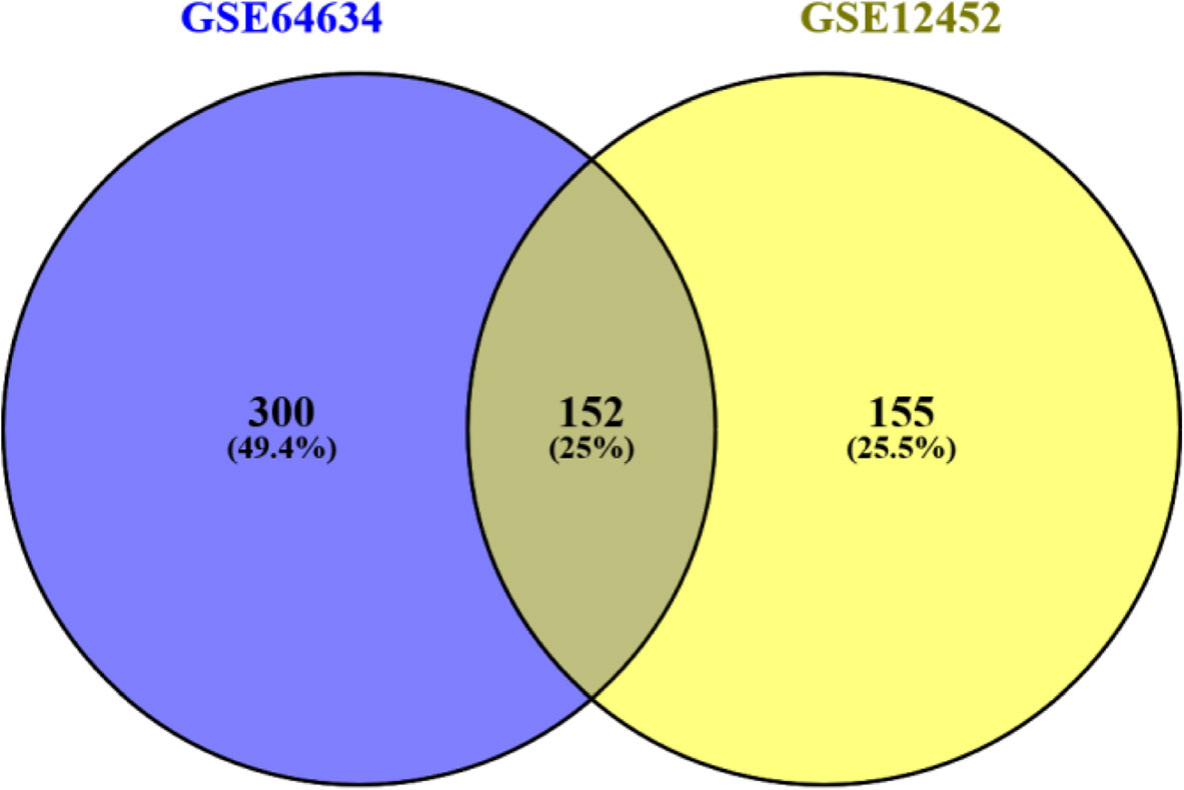
Venn diagram of 152 DEGs from the microarray datasets of GSE64634 and GSE12452

The microarray datasets of GSE64634 and GSE12452 selected from the GEO public database were uploaded to the GEO2R web-based tool to screen DEGs between normal nasopharyngeal tissues and NPC tissues. The volcano plots of DEGs were shown in Fig. 1. Each colored dot represents an up- or downregulated gene, where blue indicates genes with low levels of expression, red indicates genes with high levels of expression, and gray indicates genes with no differential expression based on the criteria of *P* value < 0.05 and |log FC| > 2.0. A total of 452 DEGs were identified from GSE64634, including 69 upregulated and 383 downregulated genes. And a total of 307 DEGs were identified from GSE12452, including 58 upregulated and 307 downregulated genes. DEG expression heatmaps of the top 50 significant genes from GSE64634 and GSE 12452 were respectively depicted in Fig. 2, and hierarchical clustering analysis showed that DEGs differed in normal nasopharyngeal and nasopharyngeal carcinoma samples. With the online tool Venny for the integrated analysis, a total of 152 genes were overlapped between GSE64634 and GSE12452, including 10 upregulated and 142 downregulated genes (Fig. 3). The results are also presented as a table of genes ordered by significance (Table 1). These 152 overlapping genes were confirmed as candidate DEGs and employed for further analysis.

### Functional and pathway enrichment analysis of DEGs

**Fig. 4:**
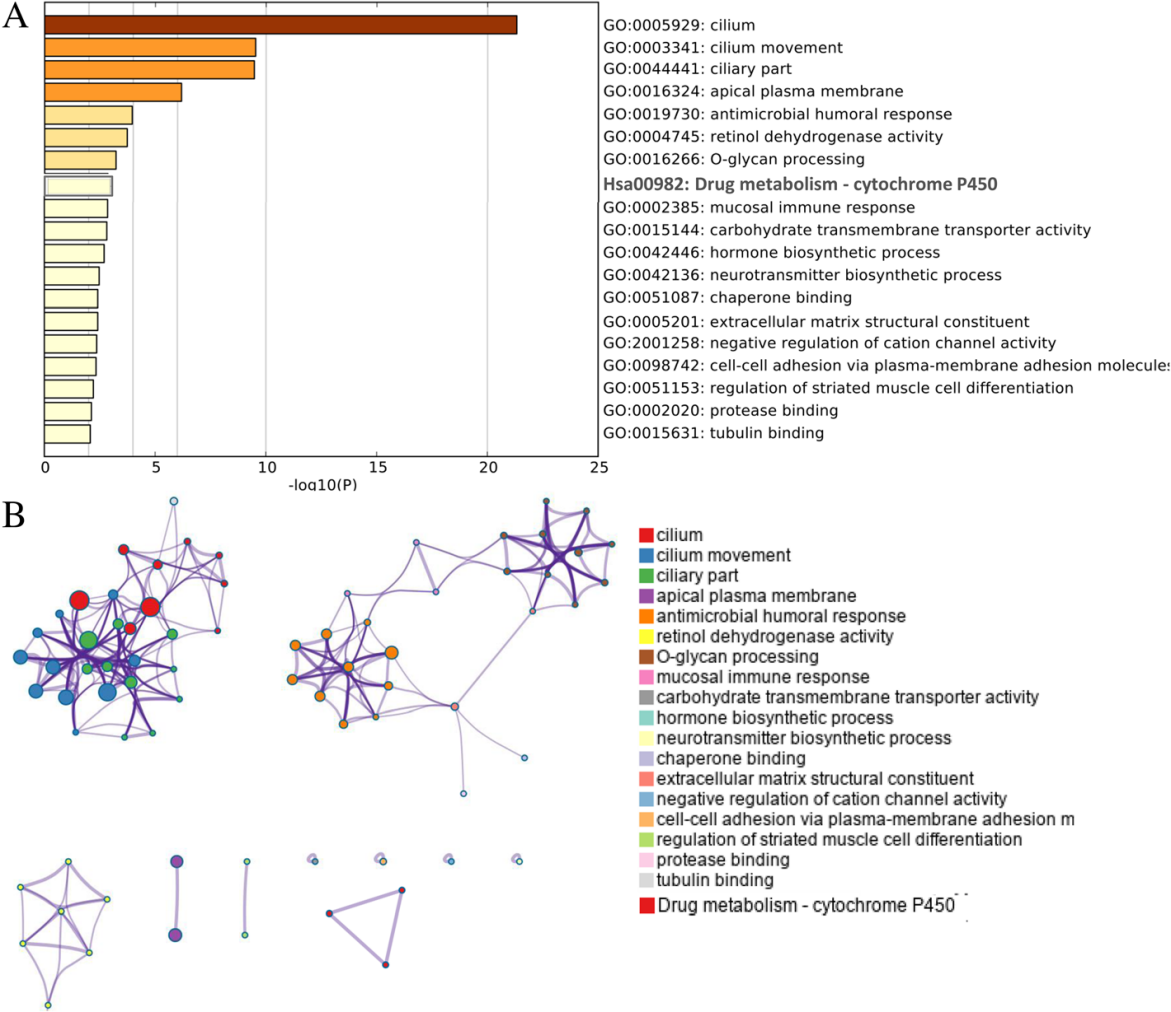
Functional and pathway enrichment analysis of DEGs. **a** GO terms and KEGG pathway were presented, and each band represents one enriched term or pathway colored according to the − log 10 *P* value. **b** Network of the enriched terms and pathways. Nodes represent enriched terms or pathways with node size indicating the number of DEGs involved in. Nodes sharing the same cluster are typically close to each other, and the thicker the edge displayed, the higher the similarity is

The GO functions and KEGG pathway enrichment analysis of candidate DEGs were performed based on the Metascape database. Terms or pathways with *P* value < 0.01, min overlap genes = 3, and min enrichment factor > 1.5 were set as the cutoff criteria. As shown in Fig. 4a, there were 18 terms and 1 pathway involved in the DEG enrichment analysis, and it indicated that these DEGs were mainly enriched in cilium, cilium movement, ciliary part, apical plasma membrane, antimicrobial humoral response, retinol dehydrogenase activity, *O*-glycan processing, mucosal immune response, carbohydrate transmembrane transporter activity, hormone biosynthetic process, neurotransmitter biosynthetic process, etc. Furthermore, these enriched terms were closely connected with each other and clustered into intact networks (Fig. 4b). KEGG pathway enrichment analysis was also displayed in Fig. 4. One significantly enriched pathway, drug metabolism-cytochrome P450, was identified with correlation with DEGs.

### PPI network construction and hub genes identification

**Fig. 5:**
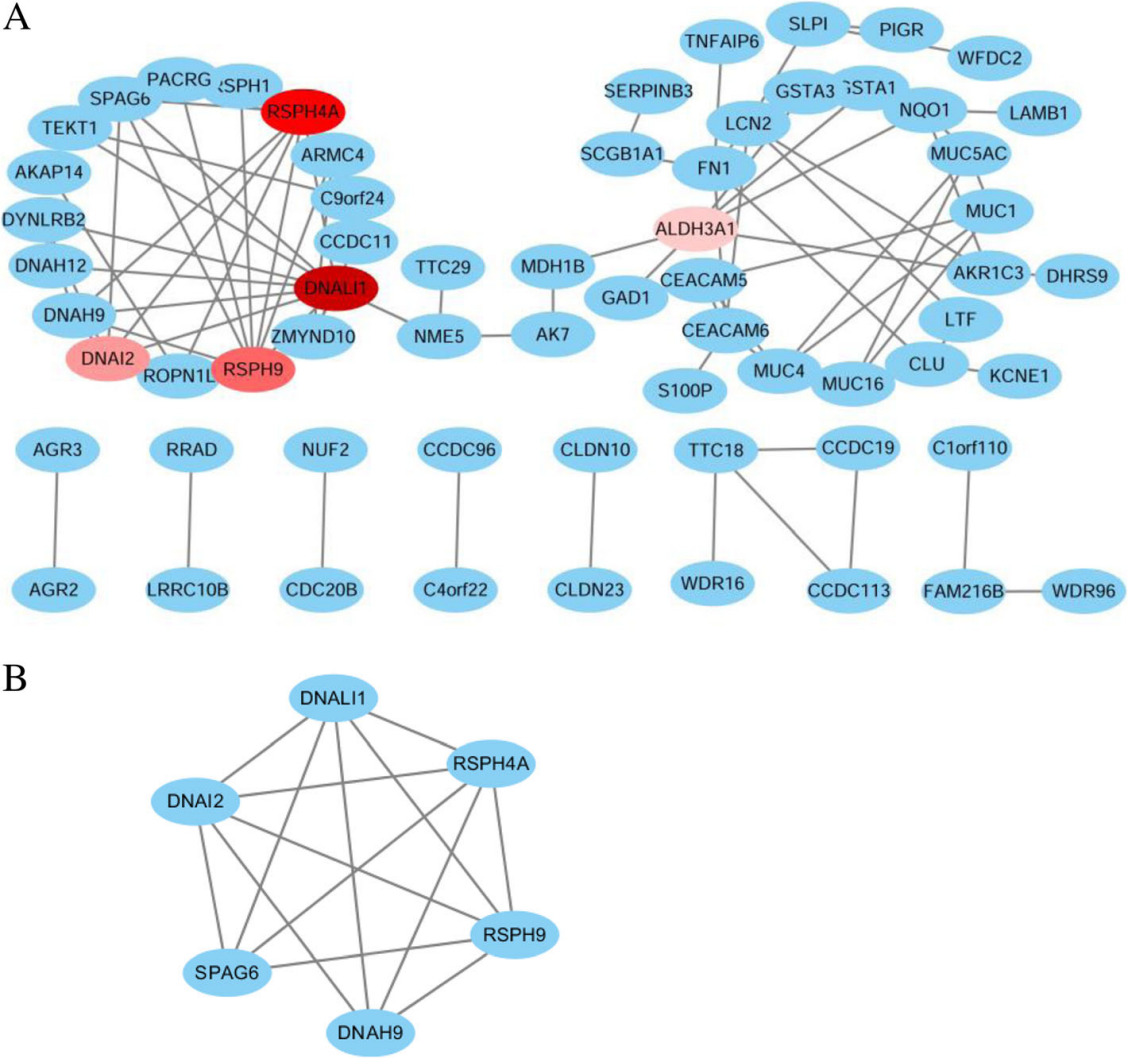
The protein-protein interaction (PPI) networks of DEGs. **a** The PPI network of total 152 DEGs. The color represents the degree of the nodes. **b** Model PPI network originated from **a** with most significant interactions (MCODE score = 5.6)

The candidate DEGs were uploaded into the online tool STRING to gain PPI information, and the result was presented as a PPI network visualized by Cytoscape (Fig. 5a). A total of 64 DEGs from 152 candidate DEGs were filtered into the network consisting of 87 interaction pairs among these 64 nodes. According to the the network topology parameters like connectivity degree, betweenness centrality, and closeness centrality, dynein axonemal light intermediate chain 1 (DNALI1; degree = 10, betweenness centrality = 0.506, closeness centrality = 0.221), radial spoke head component 4A (RSPH4A; degree = 8, betweenness centrality = 0.080, closeness centrality = 0.192), radial spoke head 9 homolog (RSPH9; degree = 8, betweenness centrality = 0.069, closeness centrality = 0.192), dynein axonemal intermediate chain 2 (DNAI2; degree = 7, betweenness centrality = 0.043, closeness centrality = 0.191), and aldehyde dehydrogenase 3 family member A1 (ALDH3A1; degree = 6, betweenness centrality = 0.554, closeness centrality = 0.264) were selected as hub DEG genes from the network,.

### Module analysis

**Fig. 6:**
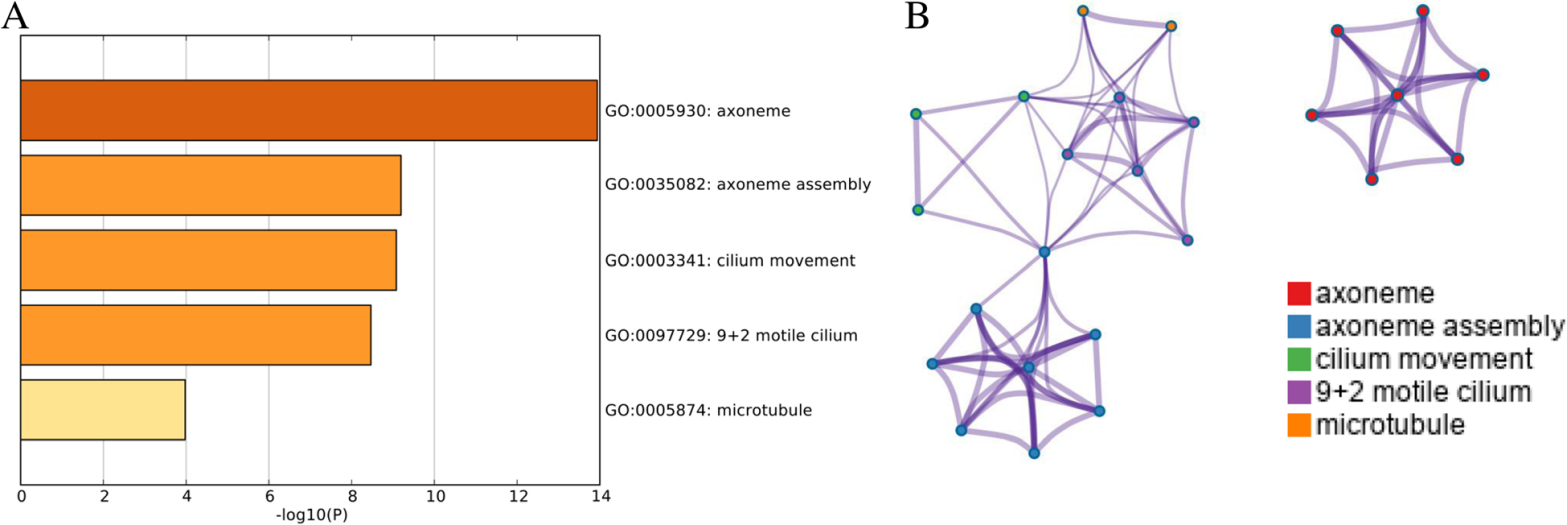
Functional and pathway enrichment analysis of the PPI module. **a** GO terms and KEGG pathway were presented, and each band represents one enriched term or pathway colored according to the − log 10 *P* value. **b** Network of the enriched terms and pathways. Nodes represent enriched terms or pathways with node size indicating the number of DEGs involved in. Nodes sharing the same cluster are typically close to each other, and the thicker the edge displayed, the higher the similarity is

Four significant models were obtained from the PPI network based on the MCODE analysis in Cytoscape. We chose the most significant module (MCODE score = 5.6) for further analysis (Fig. 5b). This module consisted of 6 nodes (DNALI1, RSPH4A, RSPH9, DNAI2, SPAG6, and DNAH9) and 14 edges, and 4 of these genes were the hub DEGs we identified. Functional enrichment analysis showed that this PPI module was significantly enriched in axoneme, axoneme assembly, cilium movement, 9 + 2 motile cilium, and microtubule (Fig. 6).

### Transcriptional factor regulatory network analysis of hub genes

**Fig. 7:**
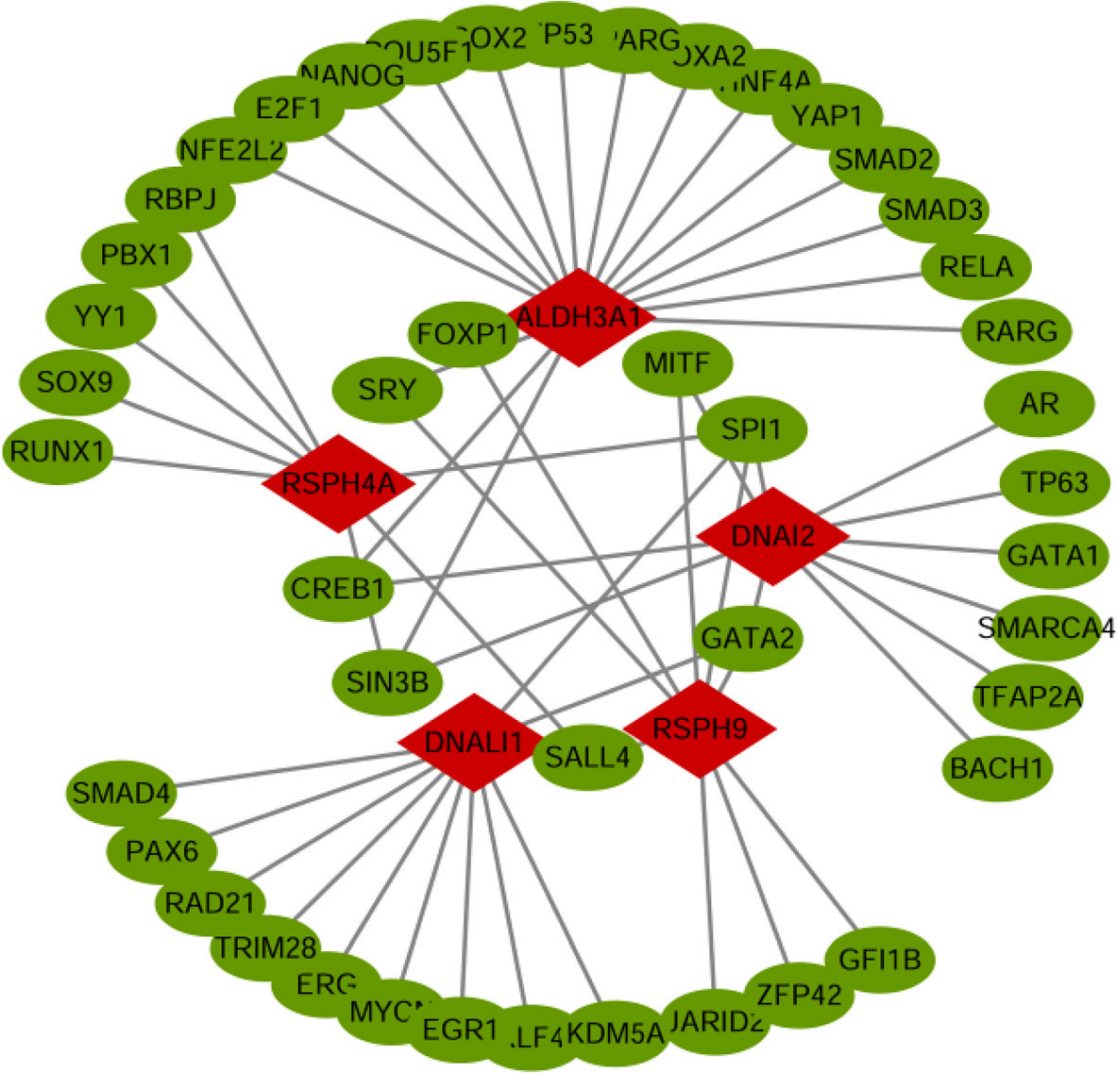
The hub gene-transcription factor (TF) regulatory network. Red diamond stands for the hub gene and green node stands for the transcription factor

For the 5 hub genes we identified, a gene-TF regulatory network was constructed including 57 interaction pairs among 5 genes and 45 TFs (Fig. 7). While DNALI1 was found to be regulated by 11 TFs, RSPH4A was regulated by 8 TFs, RSPH9 was regulated by 9 TFs, DNAI2 was regulated by 11 TFs, and ALDH3A1 was regulated by 18 TFs. In addition, various TFs were found to regulate more than one hub gene, and eight TFs were identified with a connectivity degree ≥ 2 in the gene-TF regulatory network, which means that these TFs have close interactions with these hub DEGs (Table 2). For example, spleen focus forming virus proviral integration oncogene (SPI1) was predicted to regulate both DNALI1, DNAI2, RSPH4A, and RSPH9; SIN3 transcription regulator family member B (SIN3B) was found to regulate ALDH3A1, DNAI2, and RSPH4A; and GATA binding protein 2 (GATA2) was predicted to regulate DNALI1, DNAI2, and RSPH9.

**Table 2:** The transcription factors (TFs) of hub genes

TFs	Genes	Count
SPI1	DNALI1, DNAI2, RSPH4A, RSPH9	4
SIN3B	ALDH3A1, DNAI2, RSPH4A	3
GATA2	DNALI1, DNAI2, RSPH9	3
CREB1	ALDH3A1, DNAI2	2
FOXP1	ALDH3A1, RSPH9	2
MITF	DNAI2, RSPH9	2
SALL4	RSPH4A, RSPH9	2
SRY	ALDH3A1, RSPH9	2

## Discussion

The steady improvements in disease control, survival rate, and progressive decline in the incidence of NPC have been witnessed over the past decade, but there is still a certain amount of mortality taken place in NPC patients. Moreover, the adverse effects of radiotherapy and drug toxicity or resistance to chemotherapy also are risk factors for the NPC prognosis. Considering these, increasing efforts have been made to develop personalized and sensitive treatments for NPC patient therapy [12, 13]. Precision therapy and prognosis relied on validated biomarkers with enhanced ability to screen, diagnose, and monitor tumors. Based on this, gene chip, RNA sequencing, and bioinformatic analysis have come into sight and offered a comprehensive screening of tumor biomarkers as well as a method for elucidating the underlying role of detailed biomarkers in the pathology of cancers. Over the past decade, novel approaches, such as gene therapy and molecular-targeted therapeutics, relying on scientific knowledge from the bioinformatics of cancers, have contributed to remarkable achievements and clinical benefits [14–17]. Various potential RNA and protein biomarkers have emerged based on these technologies. In this study, bioinformatic analysis was performed for NPC, and DEGs were screened from the microarray expression profiles of GSE64634 and GSE12452 from GEO database. A total of 152 DEGs were identified from the datasets, including 10 upregulated and 142 downregulated genes. Five hub DEGs (DNALI1, RSPH4A, RSPH9, DNAI2, and ALDH3A1) were obtained from the PPI network according to the network topology parameters. Furthermore, one significant module with an MCODE score = 5.6 was screened from the PPI network, consisting of four hub DEGs and SPAG6 and DNAH9.

Some earlier studies also involved bioinformatic analysis for NPC to identify candidate genes as potential biomarkers and predict progression for NPC. Chen’s research based on two gene expression profiles (GSE12452 and GSE13597) identified 179 upregulated and 238 downregulated DEGs, including 10 hub genes for which authors supposed to be useful for facilitating the early diagnosis and curative treatment of NPC [18]. Similar studies take out by An and colleagues applied GSE53819 microarray dataset for bioinformatic analysis and revealed that EXO1, CENPF, ANLN, PBK, and C15ORF42 may be involved in the mechanism of NPC via modulating the cell cycle and nucleic acid metabolic processes [19]. But in the present study, except for the dataset GSE12452, another dataset GSE64634 was also acquired for the identification of DEGs; 10 upregulated and 142 downregulated were identified based on these two datasets. Functional and pathway enrichment analysis showed that DEGs were mainly involved in cilium movement and drug metabolism-cytochrome P450 pathway, which was proved to be vital for cancer treatment. What is more, DNALI1, RSPH4A, RSPH9, DNAI2, and ALDH3A1were identified as hub DEGs from the analysis and PPI module analysis revealed that these hub genes were closely interacted thus involved the key pathway and biological processes associated with NPC.

DNALI1 (dynein axonemal light intermediate chain 1) belongs to the axonemal dynein family which is an important component of the ciliated dynamic arm and mainly responsible for the cilium movement [20]. Cilia are tiny hair-like structures on the cells in the body, and motile cilia perform an important role in the nose, ears, and airways within the lungs, working to remove unwanted inhaled particles and germs [21]. Primary ciliary dyskinesia (PCD) is an autosomal recessive or X-linked inherited disorder caused by defects in the cilium structure, and chronic rhinosinusitis (CRS) is one of the typical symptoms of PCD. The study found an abnormal expression in genes of axonemal dynein family, including DNAH5, DNAH9, DNAI1, and DNALI1, in PCD patients with immunofluorescence (IF) analysis, and mutations in these genes could cause PCD [22]. DNALI1 is also supposed as a tumor suppressor gene because of its downregulated expression and positive correlation with overall survival in breast cancer [23]. Furthermore, mutations or absences of RSPH1, RSPH4A, and RSPH9 were also found in patients with typical clinical symptoms of PCD [24]. CRS has been linked to the subsequent development of NPC. NPC patients were more likely to have a previous CRS diagnosis than normal people, and CRS was associated with greater odds of developing NPC. In addition, an integrated pathway study in the Malaysian cohort also implicated NPC in the GO axonemal dynein complex pathway [25]. Considering this, the proteins related to PCD mentioned in this study might also have a potential relationship with the occurrence of NPC. ALDH3A1 has been regarded as a therapeutic target in some cancers, like lung, hepatocellular, and prostate carcinomas, based on the evidence that abnormal levels of aldehyde dehydrogenase (ALDH) activity were expressed in human cancer types [26, 27]. Consequently, hub DEGs including DNALI1, RSPH4A, RSPH9, DNAI2, and ALDH3A1 were suggested to have key correlations with NPC.

For these DEGs, KEGG pathway analysis in the current study shown that they were enriched in drug metabolism-cytochrome P450. Cytochrome P450 (CYP450) enzymes are important for drug metabolism function in the body, and CYP450-mediated drug metabolism in cancers is crucial for cancer therapy [28]. CYP450 are implicated in phase I metabolism of 80% of drugs currently in use, including anticancer drugs, some drugs like ifosfamide and dacarbazine can be metabolized by specific CYP450 enzymes to produce intermediates with anticancer activity, and some drugs like adriamycin and etoposide with anticancer effect can be metabolized with CYP450 to enhance their anticancer activity [29]. Furthermore, CYP450 polymorphism was also found to be significantly associated with NPC susceptibility and might be a risk factor for NPC in some researches [30, 31]. Hence, the DEGs we identified involving in drug metabolism-cytochrome P450 pathway indicated that these genes might affect the activity of CYP450 enzymes as well as involved in the CYP450-mediated drug metabolism in NPC.

TFs are regulators of gene expression that are critically associated with the development and progression of human cancers. In the current study, we also identified some TFs with close interactions with hub DEGs. SPI1(or PU.1) is a member of the Ets family that is important in the regulation of hematopoiesis. Initial studies also indicate that SPI1 is a putative oncogene and has a key relationship with leukemias [32]. SIN3B serves as a scaffold for chromatin-modifying complexes that have recently been implicated in the pathogenesis of cancers. However, the function of SIN3 as a tumor suppressor or oncogene is open for debate [33]. GATA2 has critical functions in the hematopoietic system, and the mutation of GATA2 could result in hematopoietic and immune defects, leading to the occurrence of acute myelocytic leukemia. Besides, GATA2 is a pioneer transcription factor for androgen receptor (AR) in prostate cancer, and increased GATA2 and its AR-independent transactivation of IGF2 could mediate taxane resistance through the activation of IGF1/insulin receptor signaling [34]. Our results revealed that these TFs formed a connected regulatory network with hub DEGs, thus suggested that the dynamic changes in these TF activities occur in NPC cells may play important roles in regulating the expression and function of hub DEGs associated with the occurrence and progression of NPC.

The bioinformatic analysis performed in current research focused on two datasets with a total of 14 normal nasopharyngeal samples and 43 NPC samples. As a result, the sample size was limited, and high-quality studies with larger sample sizes would be included to support this study in future research. EBV is a pivotal risk factor for the pathogenesis of NPC, and most of the NPC cases are invariably associated with EBV infection. In the current study, the tumor samples included from the datasets we applied were not divided into NPC with or without EBV, so we could not elucidate the relationship between NPC with or without EBV and also could not make a comment on the molecular difference in two types of NPC. It also seems that the number of the DEGs we identified (152 DEGs) was too many, although they were differentially expressed between normal and tumor tissues, but whether all of them could be biomarkers is subject to confirmation.

## Conclusion

In conclusion, a total of 152 DEGs including 10 upregulated and 142 downregulated genes were identified from the microarray expression profiles of GSE64634 and GSE12452. Gene functional enrichment analysis indicated that these DEGs were mostly enriched in cilium movement, antimicrobial humoral response, *O*-glycan processing, mucosal immune response, hormone and neurotransmitter biosynthetic process, and drug metabolism-cytochrome P450 pathway. Five hub genes (DNALI1, RSPH4A, RSPH9, DNAI2, and ALDH3A1) and one significant module (DNALI1, RSPH4A, RSPH9, DNAI2, SPAG6, and DNAH9; MCODE score = 5.6) were obtained from the PPI network. Key TFs, such as SPI1, SIN3B, and GATA2 were also identified with close interactions with these five hub DEGs from the gene-TF network. With the integrated bioinformatic analysis, we identified the candidate DEGs of NPC and their functional and pathway enrichment as well as PPI; gene-TF networks were also clearly analyzed which indicated these hub DEGs may be potential biomarkers for NPC. However, since the sample size is limited, further studies are also required to confirm the expression and function of the identified hub DEGs in NPC.

## Abbreviations

DEGs: Differentially expressed genes
EBV: Epstein-Barr virus
GEO: Gene Expression Omnibus
GO: Gene ontology
KEGG: Kyoto Encyclopedia of Genes and Genomes
NPC: Nasopharyngeal carcinoma
PPI: Protein-protein interaction network
TFs: Transcriptional factors

## References

[1] Bray F, Ferlay J, Soerjomataram I (2018). Global cancer statistics 2018: GLOBOCAN estimates of incidence and mortality worldwide for 36 cancers in 185 countries. CA Cancer J Clin..

[2] Torre LA, Bray F, Siegel RL (2015). Global cancer statistics, 2012. CA Cancer J Clin..

[3] Zheng H, Dai W, Cheung AK (2016). Whole-exome sequencing identifies multiple loss-of-function mutations of NF-κB pathway regulators in nasopharyngeal carcinoma. Proc Natl Acad Sci USA.

[4] Henderson BE, Louie E, Soohoo JJ (1976). Risk factors associated with nasopharyngeal carcinoma. N Engl J Med..

[5] Bensouda Y, Kaikani W, Ahbeddou N (2011). Treatment for metastatic nasopharyngeal carcinoma. Eur Ann Otorhinolaryngol Head Neck Dis..

[6] Ngan HL, Wang L, Lo KW (2018). Genomic landscapes of EBV-associated nasopharyngeal carcinoma vs. HPV-associated head and neck cancer. Cancers.

[7] Tang XR, Li YQ, Liang SB, et al. Development and validation of a gene expression-based signature to predict distant metastasis in locoregionally advanced nasopharyngeal carcinoma: a retrospective, multicentre, cohort study. Lancet Oncol. 2018. 10.1016/S1470-2045(18)30080-9 29428165

[8] Wang S, Cheng Q (2006). Microarray analysis in drug discovery and clinical applications. Methods Mol Biol..

[9] Olsen LR, Campos B, Barnkob MS (2014). Bioinformatics for cancer immunotherapy target discovery. Cancer Immunol Immunother..

[10] Lou W, Chen J, Ding B (2018). Identication of invasion-metastasis-associated micro RNAs in hepatocellular carcinoma based on bioinformatic analysis and experimental validation. J Transl Med..

[11] Quackenbush J (2006). Microarray analysis and tumor classification. N Engl J Med..

[12] Shen DW, Pouliot LM, Hall MD (2012). Cisplatin resistance: a cellular self-defense mechanism resulting from multiple epigenetic and genetic changes. Pharmacol Rev..

[13] Schaue D, Mcbride WH (2015). Opportunities and challenges of radiotherapy for treating cancer. Nat Rev Clin Oncol..

[14] Cully M (2018). Targeted therapy: tipping the splicing balance to kill cancer cells. Nat Rev Cancer.

[15] Sun Y, Hofbauer JP, Harada M (2018). Cancer-type organic anion transporting polypeptide 1B3 is a target for cancer suicide gene therapy using RNA trans-splicing technology. Cancer Lett..

[16] Zhang YW, Zhong LK, Lou QW (2018). Study on the HPV-positive oropharyngeal cancer features gene based on GEO database by bioinformatic. Chin J Mod Appl Pharm.

[17] Comoglio PM, Trusolino L, Boccaccio C (2018). Known and novel roles of the MET oncogene in cancer: a coherent approach to targeted therapy. Nat Rev Cancer..

[18] Chen F, Shen C, Wang X (2017). Identification of genes and pathways in nasopharyngeal carcinoma by bioinformatics analysis. Oncotarget.

[19] An F, Zhang Z, Xia M (2015). Functional analysis of the nasopharyngeal carcinoma primary tumor associated gene interaction network. Molecular Medicine Reports.

[20] Viswanadha R, Sale WS, Porter ME. Ciliary motility: regulation of axonemal dynein motors. Cold Spring Harb Perspect Biol. 2017;9. 10.1101/cshperspect.a018325 PMC5538414 28765157

[21] Shapiro A, Davis S, Manion M (2018). Primary ciliary dyskinesia (PCD). Am J Respir Crit Care Med..

[22] Lucas JS, Barbato A, Collins SA, et al. European Respiratory Society guidelines for the diagnosis of primary ciliary dyskinesia. Eur Respir J. 2017;49(1). 10.1183/13993003.01090-2016 PMC6054534 27836958

[23] Parris TZ, Danielsson A, Nemes S (2010). Clinical implications of gene dosage and gene expression patterns in diploid breast carcinoma. Clin Cancer Res..

[24] Frommer A, Hjeij R, Loges NT (2015). Immunofluorescence analysis and diagnosis of primary ciliary dyskinesia with radial spoke defects. Am J Respir Cell Mol Biol..

[25] Chin YM, Ping TL, Abdul AN (2016). Integrated pathway analysis of nasopharyngeal carcinoma implicates the axonemal dynein complex in the Malaysian cohort. Int J Cancer..

[26] Counihan JL, Wiggenhorn AL, Anderson KE, Nomura DK (2018). Chemoproteomics-enabled covalent ligand screening reveals ALDH3A1 as a lung cancer therapy target. ACS Chem Biol..

[27] Patel M, Lu L, Zander DS (2008). ALDH1A1 and ALDH3A1 expression in lung cancers: correlation with histologic type and potential precursors. Lung Cancer..

[28] Zhang YW, Fang L, Zheng XW (2017). Study advance on epigenetic regulation of cytochrome p450 enzymes. Chin J Mod Appl Pharm.

[29] Mittal B, Tulsyan S, Kumar S (2015). Cytochrome p450 in cancer susceptibility and treatment. Advances in clinical chemistry.

[30] Tiwawech D, Srivatanakul P, Karalak A (2006). Cytochrome P450 2A6 polymorphism in nasopharyngeal carcinoma. Cancer Letters.

[31] Hildesheim A, Anderson LM (1997). CYP2E1 genetic polymorphisms and risk of nasopharyngeal carcinoma in Taiwan. J Natl Cancer Inst.

[32] Moreau-Gachelin F, Tavitian A (1988). Spi-1 is a putative oncogene in virally induced murine erythroleukaemias. Nature..

[33] Lewis MJ, Liu J, Libby EF (2016). SIN3A and SIN3B differentially regulate breast cancer metastasis. Oncotarget..

[34] Plymate SR, Bhatt RS (2015). Taxane resistance in prostate cancer mediated by AR-independent GATA2 regulation of IGF2. Cancer Cell..

